# Associations between long-term conditions and upper gastrointestinal cancer incidence: A prospective population-based cohort of UK Biobank participants

**DOI:** 10.1177/26335565211056136

**Published:** 2021-11-17

**Authors:** Jennifer Marley, Barbara I Nicholl, Sara Macdonald, Frances S Mair, Bhautesh D Jani

**Affiliations:** 1 General Practice and Primary Care, Institute of Health and Wellbeing, College of Medical, Veterinary and Life Sciences, University of Glasgow, UK

**Keywords:** Oesophageal cancer, Stomach cancer, Comorbidity, Long-term conditions, Cancer, Incidence

## Abstract

**Background/Aims:**

Upper gastrointestinal cancers (oesophageal/stomach) have high mortality rates and are often diagnosed after the disease has progressed, making it important to identify populations at greater risk of upper gastrointestinal (UGI) cancer to promote earlier diagnosis. This study aims to determine if there is an association between a broad range of long-term conditions (LTCs) and incidence of UGI cancers.

**Method:**

A prospective-based cohort of 487,798 UK Biobank participants (age 37–73 years) after excluding previous UGI cancer. Least Absolute Shrinkage and Selection Operator (LASSO) regression used to identify candidate LTCs as predictors for UGI cancer. Strength of association was studied using Cox’s regression adjusting for demographics and lifestyle factors.

**Results:**

After median follow-up period of 86 months, 598 participants developed oesophageal cancer; 397 developed stomach cancer. In fully adjusted models, participants with alcohol addiction (Hazard Ratio-HR 4.11, 95% Confidence Interval-CI 2.01–8.43), Barrett’s oesophagus (HR 5.68, 95% CI 3.36–9.58), bronchiectasis (HR 2.72, 95% CI 1.01–7.31), diabetes (HR 1.38, 95% CI 1.06–1.81), hiatus hernia (HR 1.69, 95% CI 1.16–2.45), Parkinson’s disease (HR 3.86, 95% CI 1.60–9.37) and psoriasis/eczema (HR 1.53, 95% 1.08–2.17) were observed to have a higher risk of oesophageal cancer. Stomach cancer incidence was higher among participants with anorexia/bulimia (HR 8.86, 95% CI 1.20–65.14), Barrett’s oesophagus (HR 3.37, 95% 1.39–8.14), chronic fatigue syndrome (HR 3.36, 95% CI 1.25–9.03), glaucoma (HR 2.06, 95% CI 1.16–3.67), multiple sclerosis (HR 4.60, 95% CI 1.71–12.34), oesophageal stricture (HR 1.04, 95% CI 1.46–74.46) and pernicious anaemia (HR 6.93, 95% CI 3.42–14.03).

**Conclusion:**

Previously unrecognised LTCs may have a role in symptom appraisal and risk assessment of UGI cancer in primary care. Further research should explore mechanisms underpinning these findings and determine whether they are replicable in other populations.

## Introduction

By 2035, it is projected that more than 500,000 people will be diagnosed with cancer each year in the UK.^1^ Understandably, cancer research has remained a national clinical priority for the UK Government and the National Health Service (NHS). Strategies such as the Scottish Cancer Taskforce (SCT) and the NHS Long-Term Plan (LTP) aim to improve cancer outcomes through earlier detection, better diagnostic methods and advanced treatment therapies at population levels.^2-3^ Long-term conditions (LTCs) and cancer share common risk factors, for example, unhealthy lifestyle factors and increasing age.^4-5^ There is also evidence that specific LTCs can predispose to certain cancers, although many cancer prevention strategies focus on lifestyle risk factors, rather than considering the risk of LTCs.^4-8^

In the UK, upper gastrointestinal cancers (oesophageal and stomach) account for a small number of new cancer cases per year and share many risk factors, such as alcohol consumption, smoking and obesity. ^4-5^ However, patients are often diagnosed when the cancer has advanced which could explain the high mortality rate of upper gastrointestinal (UGI) cancers. ^4-5^

Previous studies have demonstrated associations between UGI cancer and a limited number of LTCs such as Barrett’s oesophagus and Helicobacter pylori *(*H. pylori*)* infection. ^9-11^ This study examines associations, if any, between UGI cancer and 50 LTCs. Further investigation into the associations, if any, of a wider number of LTCs and UGI cancer incidence may have implications for the symptom appraisal and diagnosis of UGI cancer in primary care.

### Aims and objectives

This study aims to investigate the relationship between a wide range of LTCs and the incidence of oesophageal and stomach cancers and to identify specific LTCs, if any, associated with a higher incidence of UGI cancers.

## Methods

### Study design and participants

This research has been conducted using UK Biobank, approved project number 14151. The UK Biobank has full ethical approval from the NHS National Research Ethics Service (16/NW/0274).

**Figure 1.**
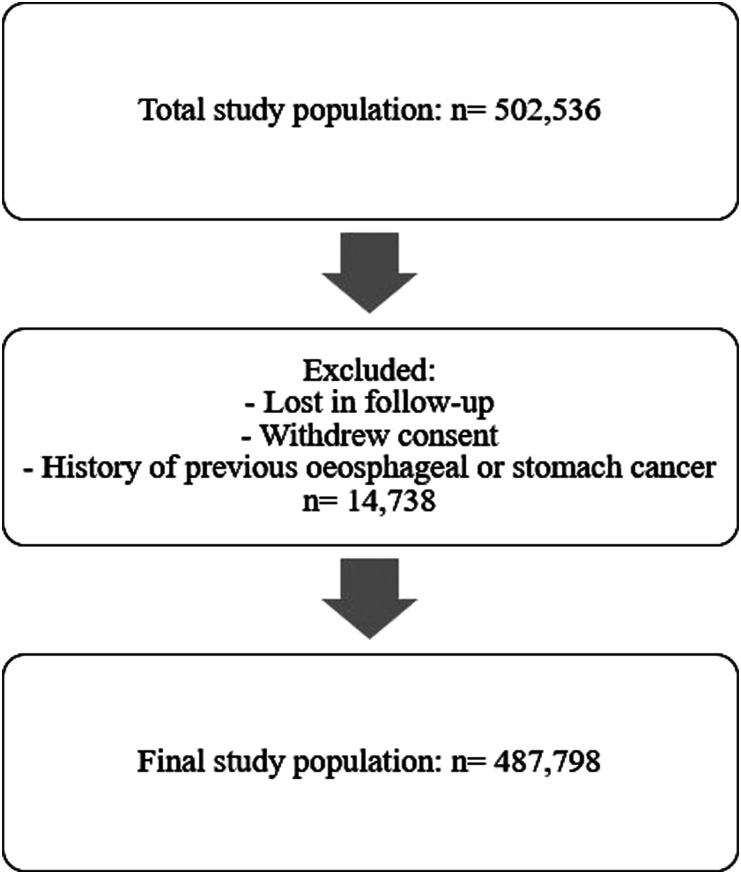
Flowchart of the study population. Study population was reduced following the exclusion criteria.

This study was a prospective population-based cohort, that examined data from the UK Biobank cohort. The sample size for the analysis included 487,798 participants, see Figure 1. Participant data were excluded if they were lost during the follow-up, withdrew consent or had a history of previous UGI cancers. Participants were volunteers recruited between 2006 and 2010 and were aged between 37–73 years

### Exposure variables

At baseline, a self-reported detailed account of sociodemographic, lifestyle and medical information was collected from all participants. We considered a list of 50 LTCs (see appendix Table A1) – including conditions already linked to UGI cancer, such as Barrett’s oesophagus and H. pylori infection. We expanded our list used in previous research on multimorbidity in UK Biobank to include additional LTCs associated with UGI cancer.^12^ Age was described as a continuous variable, and sex was characterised as a categorical variable. Socioeconomic status was based upon the Townsend score (an area-based measure of deprivation in the UK) and classified into quintiles: category 1 being the least deprived and category 5 being the most deprived. ^13-14^ The body mass index (BMI) of participants was categorised using the current World Health Organisation’s ranges: category 1 described a BMI of less than 18.5, category 2: 18.5–25, category 3: 25–30, category 4: 30–35 and category 5 was a BMI of more than 40. ^15^ Physical activity was self-reported and classified as none (no physical activity in the last 4 weeks), low (light activity only in the last 4 weeks), medium (heavy and/or walking for pleasure and/or other exercises in the last 4 weeks) and high (strenuous sports in the last 4 weeks).^16^ Alcohol consumption was recorded in weekly units and categorised as follows: category 1: 1–14 units (sensible), category 2: 15–35 units in females and 15–50 units in males (hazardous) and category 3: 35 units or above in females and 50 or above units in males (harmful). ^17^ Smoking status was categorised as never smoked, previous smoker and current smoker. A clinical quantification of cigarette smoking, described as ‘pack years’, was used to assess a person’s lifetime exposure to tobacco. Pack years were calculated by multiplying the number of packs of cigarettes a person smoked per day by the number of years the person has smoked.^18^

### Outcome variables

The duration of the follow-up period ranged between 78–94 months, and the median period was 86 months. Follow-up data included the diagnosis of oesophageal or stomach cancer. Linked data from cancer registries were provided by UK Biobank to study cancer incidence in the study population. Oesophageal cancer incidence was classified according to the International Classification of Diseases, 10th Revision (ICD-10) code C15 and stomach cancer C16, respectively.^19^

### Statistical analysis

All statistical analyses were performed using the R statistical software version 4.0.2. The distribution of demographic and lifestyle factors in the sample was described using mean and standard deviation for the continuous variables and percentages for the categorical variables. We used one way ANOVA test and Chi-square test to assess the differences between groups, those with and without oesophageal and stomach cancer, respectively.

The initial analysis of data involved using all eligible data provided by the UK Biobank cohort and was split into two stages: variable selection and survival analysis. The diagnosis of either oesophageal or stomach cancer were the outcome variables.

A penalised regression method known as Least Absolute Shrinkage and Selection Operator (LASSO) regression was used to reduce the dimension of the data and variable selection of candidate LTCs. LASSO regression reduces the complexity of the model by penalising the regression model to shrink the regression coefficients towards zero.^20^ This method utilised the R packages; glmnet’ and ‘Matrix’.^21 and 22^

The second phase of analysis was survival analysis using Cox’s proportional hazard regression models. The models were adjusted for age, sex, socioeconomic status, BMI, level of physical activity, alcohol consumption and smoking status. The relationship between the predictor variables selected by LASSO regression and the outcome variable was tested in fully adjusted Cox’s proportional hazard models. Results were reported as hazard ratios (HRs) with 95% confidence intervals (CI). Statistical significance was indicated from a *p*-value of less than 0.05. The survival analysis was performed using the R packages ‘survival’, ‘foreach’ and ‘ggplot2’.^23-25^ The survival models were tested for multicollinearity using the variance inflation factor (VIF) values for all predictor variables, using the R package ‘car’.^26^

## Results

Of the 502,536 participants recruited to UK Biobank between 2006 and 2010, a total of 487,798 (97%) participants were included in this study, after excluding those with previous UGI cancer and missing variables.

Table 1 compares the demographic characteristics of participants that developed UGI cancer and those that did not. The mean age of participants was 57 years, and the range was 37–73 years. After 86 months of the median follow-up period, 598 participants developed oesophageal cancer and 397 participants developed stomach cancer. The proportion of participants that developed UGI cancer was similar across all socioeconomic backgrounds. This study considered a total of 50 LTCs as candidate risk factors of oesophageal cancer and stomach cancer – see appendix Table A1.

**Table 1.** Participant characteristics for those with and without oesophageal and stomach cancer.

Demographics	Participants with oesophageal cancer (n = 598)	Participants without oesophageal cancer (n = 487,200)	*p*-value for difference	Participants with stomach cancer (n = 397)	Participants without stomach cancer (n = 487,401)	*p*-value for difference
**Age (years), mean (SD)**	62 (6)	57 (8)	<0.01	62 (7)	57 (8)	<0.01
**Sex**						
Male	438 (73.5%)	221,834 (45.5%)	<0.01	282 (70.7%)	221,990 (54.4%)	<0.01
Females	160 (26.5%)	265,366 (54.5%)		115 (29.3%)	265,411 (45.6%)	
**Townsend score**						
Quintile 1	103 (16.8%)	98,651 (20.1%)	<0.01	64 (15.6%)	98,690 (20.1%)	0.06
Quintile 2	113 (18.5%)	97,871 (20%)		72 (18.1%)	97,912 (19.9%)	
Quintile 3	120 (20.1%)	97,715 (20%)		79 (20.2%)	97,756 (20%)	
Quintile 4	110 (18.1%)	97,482 (20%)		86 (21.7%)	97,506 (20%)	
Quintile 5	152 (26.5%)	95,481 (19.9%)		96 (24.3%)	95,537 (20%)	
**BMI category**						
1: <18.5	7 (1.1%)	2494 (0.5%)	<0.01	2 (0.7%)	2499 (0.5%)	<0.01
2: 18.5–25	122 (20.1%)	154,024 (31.6%)		90 (22.7%)	154,056 (31.5%)	
3: 25–30	279 (46.8%)	209,377 (42.9%)		180 (45.2%)	209,476 (42.9%)	
4: 30–35	139 (23.2%)	87,131 (17.9%)		78 (20%)	87,192 (17.9%)	
5: 35–40	34 (5.8%)	24,729 (5.1%)		35 (8.5%)	24,728 (5.2%)	
6: >40	17 (3%)	9445 (2%)		12 (2.9%)	9450 (2%)	
**Level of physical activity**						
High	40 (6.5%)	50,019 (10.1%)	<0.01	37 (9.1%)	50,022 (10.1%)	<0.01
Medium	478 (78.4%)	392,818 (79.5%)		305 (74.7%)	392,991 (79.5%)	
Low	42 (6.9%)	18,871 (3.8%)		31 (7.6%)	18,882 (3.8%)	
None	50 (8.2%)	32,765 (6.6%)		35 (8.6%)	32,780 (6.6%)	
**Alcohol consumption (weekly units)**						
1	315 (53.1%)	309,461 (63.6%)	<0.01	213 (54.1%)	309,563 (63.6%)	
2	200 (33.3%)	147,593 (30.2%)		148 (36.8%)	147,645 (30.2%)	
3	83 (13.6%)	30,146 (6.2%)		36 (9.1%)	30,193 (6.2%)	
**Smoking status**						
Never	184 (31.1%)	267,421 (54.8%)	<0.01	148 (37.1%)	267,457 (54.8%)	
Previous	301 (50.3%)	168,837 (34.6%)		181 (45.2%)	168,957 (34.6%)	
Current	113 (18.6%)	50,942 (10.6%)		68 (17.7%)	50,987 (10.6%)	

Standard deviation (SD).

Townsend score: Quintile 1: least deprived and Quintile 5: most deprived.

Body Mass Index (BMI). Category 1 described a BMI of less than 18.5, category 2: 18.5–25, category 3: 25–30, category 4: 30–35 and category 5 was a BMI of more than 40.

Level of physical activity was described as high, medium, low or none.

Alcohol consumption: category 1: 1–14 units (sensible), category 2: 15–35 units in females and 15–50 units in males (hazardous) and category 3: >35 units in females and >50 units in males (harmful).

Variable selection with LASSO regression identified 22 LTCs and LTC count as candidate risk factors for oesophageal cancer. The full list of 22 LTCs identified can be found in the appendix Table A2. Seven LTCs were found to have a statistically significant association with UGI cancer after adjusting for age, sex, socioeconomic status, BMI, level of physical activity, alcohol consumption and smoking status. The seven LTCs with significant associations with oesophageal cancer were the following: alcohol addiction (HR 4.11, 95% CI 2.01–8.43, *p* < 0.01), Barrett’s oesophagus (HR 5.68, 95% CI 3.36–9.58, *p* < 0.01), bronchiectasis (HR 2.72, 95% CI 1.01–7.31, *p* = 0.05), diabetes (HR 1.38, 95% CI 1.06–1.81, *p* = 0.02), hiatus hernia (HR 1.69, 95% CI 1.16–2.45, *p* < 0.01), Parkinson’s disease (HR 3.86, 95% CI 1.60–9.37, *p* < 0.01) and psoriasis/eczema (HR 1.53, 95% CI 1.08–2.17, *p* = 0.02).

Variable selection also identified 27 LTCs and LTC count as candidate risk factors for stomach cancer, – see appendix Table A2. After adjusting for the same demographic and lifestyle factors, survival analysis identified the following significant LTCs as predictor variables for stomach cancer shown in Table 2:anorexia/bulimia (HR 8.86, 95% CI 1.20–65.14, *p* = 0.03), Barrett’s oesophagus (HR 3.37, 95% CI 1.39–8.14, *p* < 0.01), chronic fatigue syndrome (HR 3.36, 95% CI 1.25–9.03, *p* = 0.02), glaucoma (HR 2.06, 95% CI 1.16–3.67, *p* = 0.01), multiple sclerosis (HR 4.60, 95% CI 1.71–12.34, *p* < 0.01), oesophageal stricture (HR 1.04, 95% CI 1.46–74.46, *p* = 0.02) and pernicious anaemia (HR 6.93, 95% CI 3.42–14.03, *p* < 0.01). VIF values for all predictor variables in the survival models above were calculated to assess for multicollinearity. All VIF values were found to be <5 in both survival models (please see Appendix Table A3).

**Table 2.** Variable selection and survival analysis results for candidate risk factors for oesophageal and stomach cancer.

Oesophageal cancer candidate risk factors		
LTCs	Hazard ratios (95% CI)	*p*-value
Alcohol addiction	4.11 (2.01–8.43)	<0.01
Barrett’s oesophagus	5.68 (3.36–9.58)	<0.01
Bronchiectasis	2.72 (1.01–7.31)	0.05
Diabetes	1.38 (1.06–1.81)	0.02
Hiatus hernia	1.69 (1.16–2.45)	<0.01
Parkinson’s disease	3.86 (1.60–9.37)	<0.01
Psoriasis/eczema	1.53 (1.08–2.17)	0.02
Stomach cancer candidate risk factors		
**LTCs**	**Hazard ratios (95% CI)**	*p* **-value**
Anorexia/Bulimia	8.86 (1.20–65.14)	0.03
Barrett’s oesophagus	3.37 (1.39–8.14)	<0.01
Chronic fatigue syndrome	3.36 (1.25–9.03)	0.02
Glaucoma	2.06 (1.16–3.67)	0.01
Multiple sclerosis	4.60 (1.71–12.34)	<0.01
Oesophageal stricture	1.04 (1.46–74.46)	0.02
Pernicious anaemia	6.93 (3.42–14.03)	<0.01

As shown in Table 3, oesophageal cancer and stomach incidence was greater for participants with the LTCs described above as compared to participants without these LTCs, respectively.

**Table 3.** Proportion of participants who developed UGI cancer with and without an LTC identified as a candidate risk factor.

LTC status	Number of participants	Number of participants that developed oesophageal cancer	LTC status	Number of participants	Number of participants that developed stomach cancer
With an alcohol addiction	741	8 (1.1%)	With anorexia/bulimia	360	1 (0.3%)
Without an alcohol addiction	487,057	590 (0.1%)	Without anorexia/bulimia	487,438	396 (0.08%)
With Barrett’s oesophagus	1420	15 (1.1%)	With Barrett’s oesophagus	1420	5 (0.4%)
Without Barrett’s oesophagus	486,378	583 (0.1%)	Without Barrett’s oesophagus	486,378	392 (0.08%)
With bronchiectasis	1101	4 (0.4%)	With CFS	2092	4 (0.2%)
Without bronchiectasis	486,697	594 (0.1%)	Without CFS	485,706	393 (0.08%)
With diabetes	24,238	67 (0.3%)	With glaucoma	5174	12 (0.2%)
Without diabetes	463,560	531 (0.1%)	Without glaucoma	482,624	385 (0.08%)
With hiatus hernia	10,827	30 (0.3%)	With MS	1486	4 (0.3%)
Without hiatus hernia	476,971	568 (0.1%)	Without MS	486,312	393 (0.08%)
With Parkinson’s disease	808	5 (0.6%)	With oesophageal stricture	103	1 (1%)
Without Parkinson’s disease	486,990	593 (0.1%)	Without oesophageal stricture	487,695	396 (0.08%)
With psoriasis/eczema	17,470	34 (0.2%)	With pernicious anaemia	1478	8 (0.5%)
Without psoriasis/eczema	470,328	564 (0.1%)	Without pernicious anaemia	486,320	389 (0.08%)

Long-term conditions (LTCs).

## Discussion

In this prospective population-based cohort, significant associations were found between several LTCs and higher incidence of oesophageal and stomach cancers.

Our findings suggest certain LTCs may play a role in the symptom appraisal and risk assessment of oesophageal and stomach cancer, particularly previously unrecognised conditions such as bronchiectasis, chronic fatigue syndrome, diabetes, glaucoma, multiple sclerosis, Parkinson’s disease and psoriasis/eczema. Future research should be directed towards better understanding the mechanisms underpinning these associations and exploring to what extent these findings are replicable in different populations. A better understanding of the relationships between the identified LTCs and UGI cancers could potentially contribute to the process of symptom appraisal for both patients and primary care physicians. Currently, investigations in UGI cancer are initiated based on age and presence of alarm symptoms. ^27-28^ Greater understanding of the role of the LTCs identified in this paper may improve risk assessment of UGI cancer patients and expedite the investigation process, which in turn may lead to earlier diagnosis of UGI cancers and improved survival for patients.

### Strengths and limitations

Strengths of this study include the wide range of LTCs examined as risk factors – including conditions with and without established links to UGI cancer, the large sample size and prospective design of the study. We were also able to adjust for a wide range of potential confounding factors.

There are, however, several limitations. There is a possibility of selection bias as the UK Biobank population is less ethnically diverse and less socioeconomically deprived than the general UK population.^12^ UK Biobank provides self-reported information on LTCs, which is another potential limitation. However, several studies have investigated the validity of self-reported data and have found the data obtained is reasonably accurate.^29-31^ We acknowledge that the number of participants with an identified chronic disease and those who developed oesophageal cancer or stomach cancer in this study was small; however, this can be accounted for by the rarity of the forms of cancer. Finally, the information on presence of UGI cancer related symptoms among participants was not available which is likely to influence the relationship between LTCs and UGI cancer.

### Comparison with existing literature

The annual incidence of oesophageal cancer and stomach cancer for the UK population is 14.9 per 100,000 and 10.3 per 100,000, respectively. ^32^ Our study observed a similar event rate: 15.3 per 100,000 for oesophageal cancer and 10.2 per 100,000 for stomach cancer annually, suggesting the study population is similar to the UK general population in terms of prevalence of these cancers.

Associations between some LTCs and cancer have been previously investigated; however, to the best of our knowledge, this study is the first to investigate associations across such a wide range of LTCs and oesophageal and stomach cancer. There have been several attempts to explain possible mechanisms that may link long-term conditions to UGI cancer; however, the findings were not conclusive.^33-41^ A meta-analysis by Xu and colleagues produced similar results to that reported in our study in relation to diabetes and oesophageal cancer: risk ratio of 1.28, 95% CI 1.12–1.47 and suggested hyperglycaemia and oxidative stress interactions may provide a mechanism linking diabetes and oesophageal cancer. ^33^ An increase in overall cancer risk has been reported among patients with multiple sclerosis and has been linked to immunomodulating or immunosuppressive therapies. ^34-36^ However, there are inconsistencies in study design and populations used in these reports, for example, Fois and colleagues did not consider patients’ cancer history.^35^ Genetic associations common to both Parkinson’s disease and cancer have been hypothesized to play a role in an increase in a patient’s overall cancer risk following diagnosis of Parkinson’s disease.^37^ However, data regarding associations between Parkinson’s disease and oesophageal cancer incidence is inconsistent. One study demonstrated an increased relative risk of oesophageal cancer: 1.09, 95% CI 0.65–1.83, although our results showed a much greater effect size.^38^ In contrast, Goldacre found a lower risk of oesophageal cancer among patients with Parkinson’s disease: relative risk of 0.85, 95% CI 0.78–0.93.^39^ Our results are reflective of a study by Trafford and colleagues who found the relative risk of oesophageal cancer among patients with psoriasis was 2.05, 95% CI 1.04–4.07. Similarly, D’Arcy and colleagues found an adjusted odds ratio of 1.29, 95% CI 1.08–1.54 for oesophageal cancer among participants with eczema.^40-41^

Our findings are in keeping with numerous reports of associations between oesophageal cancer and alcohol addiction and hiatus hernia, and stomach cancer and anorexia/bulimia, oesophageal stricture and pernicious anaemia, as well as both UGI cancers and Barrett’s oesophagus.^42-47^ Surprisingly, this study did not find a significant association between H. pylori infection and UGI cancer as has been reported in previous literature.^11^ This may be attributed to participants underreporting the condition.

## Conclusion

This study observed a higher incidence of UGI cancer among participants with LTCs not previously recognised to have associations with this form of cancer. Participants with bronchiectasis, diabetes, Parkinson’s disease and psoriasis/eczema demonstrated a greater risk of oesophageal cancer. While participants with chronic fatigue syndrome, glaucoma and multiple sclerosis observed a greater risk of stomach cancer.

Further research needs to be undertaken to determine if these results are replicable in different populations and to determine the nature of the relationship of these conditions with UGI incidence. If these associations are found to be consistent in different populations, several additional LTCs previously not associated with UGI cancer may have a role in symptom appraisal and risk assessment of oesophageal and stomach cancer in primary care. This may lead to earlier investigation and diagnosis of patients with UGI cancer. This is important as currently oesophageal and stomach cancers are often diagnosed at later stages and have a poor survival rate.

## Appendix

**Table A1.** Candidate risk factors for oesophageal and stomach cancer. Table 1 lists the candidate risk factors for oesophageal and stomach cancer. These LTCs were obtained from participant information collected at the beginning of this study.

Alcohol addiction	Anorexia/bulimia	Anxiety	Asthma	Atrial fibrillation
Barrett’s oesophagus	Bronchiectasis	Chronic fatigue syndrome	Chronic kidney disease	Chronic liver disease
Chronic obstructive pulmonary disease	Chronic sinusitis	Constipation	Coronary heart disease	Dementia
Depression	Diabetes	Diverticular disease	Duodenal ulcer	Endometriosis
Epilepsy	Gastritis	Glaucoma	Heart failure	*Helicobacter pylori* infection
Hiatus hernia	Hypertension	Indigestion	Inflammatory bowel disease	Irritable bowel syndrome
Meniere’s disease	Migraine	Multiple sclerosis	Oesophageal stricture	Osteoporosis
Painful condition	Parkinson’s disease	Peripheral vascular disease	Pernicious anaemia	Polycystic ovary syndrome
Prostate diseases	Psoriasis/eczema	Psychoactive disorders	Pyloric stenosis	Rheumatoid arthritis
Schizophrenia/bipolar disorder	Stomach ulcer	Stroke/transient ischaemic attack	Thyroid diseases	Viral hepatitis

**Table A2.** Variable selection and survival analysis results for candidate risk factors for oesophageal and stomach cancer. Table 2 lists the candidate risk factors for oesophageal and stomach cancer identified using variable selection and survival analysis.

Oesophageal and stomach cancer candidate risk factors		
LTCs	Hazard ratios (95% CI)	*p*-value
**Alcohol addiction**	**4.11 (2.01–8.43)**	**<0.01**
Anxiety	0.53 (0.18–1.07)	0.16
Asthma	1.27 (1.00–1.60)	0.05
**Barrett’s oesophagus**	**5.68 (3.36–9.58)**	**<0.01**
**Bronchiectasis**	**2.72 (1.01–7.31)**	**0.05**
Chronic heart disease	0.75 (0.55–1.03)	0.07
Chronic liver disease	1.71 (0.54–5.41)	0.36
Chronic obstructive pulmonary disease	1.14 (0.75–1.73)	0.54
Dementia	6.27 (0.88–44.71)	0.07
**Diabetes**	**1.38 (1.06–1.81)**	**0.02**
Diverticular disease	0.53 (0.22–1.28)	0.16
Epilepsy	0.19 (0.03–1.35)	0.10
*Helicobacter* pylori infection	0.43 (0.06–3.05)	0.40
**Hiatus hernia**	**1.69 (1.16–2.45)**	**<0.01**
Hypertension	2.11 (0.93–1.33)	0.23
Indigestion	2.37 (0.59–9.53)	0.22
Meniere’s disease	1.65 (0.53–5.15)	0.38
Multiple sclerosis	1.35 ×10^−6^ (0-inf*)	0.99
**Parkinson’s disease**	**3.86 (1.60–9.37)**	**<0.01**
Pernicious anaemia	2.17 (0.81–5.81)	0.12
**Psoriasis/eczema**	**1.53 (1.08–2.17)**	**0.02**
Stomach ulcer	1.55 (0.85–2.83)	0.15
LTC count	1.07 (0.96–1.20)	0.23
Stomach cancer candidate risk factors		
LTCs	Hazard ratios (95% CI)	*p*-value
Alcohol addiction	2.00 ×10^−7^ (0-inf*)	0.99
**Anorexia/bulimia**	**8.86 (1.20–65.14)**	**0.03**
Anxiety	0.58 (0.22–1.55)	0.28
Atrial fibrillation	1.72 (0.85–3.47)	0.13
**Barrett’s oesophagus**	**3.37 (1.39–8.14)**	**0.01**
Bronchiectasis	3.10 ×10^−7^ (0-inf*)	0.99
**Chronic fatigue syndrome**	**3.36 (1.25–9.03)**	**0.02**
Chronic heart disease	1.07 (0.76–1.51)	0.68
Chronic obstructive pulmonary disease	0.54 (0.25–1.15)	0.11
Diabetes	1.38 (1.00–1.91)	0.05
Duodenal ulcer	0.36 (0.05–2.59)	0.31
Gastritis	2.29 ×10^−7^ (0-inf*)	1.00
**Glaucoma**	**2.06 (1.16–3.67)**	**0.01**
Heart failure	1.12x10^−7^ (0-inf*)	1.00
*Helicobacter* pylori infection	2.06x10^−7^ (0-inf*)	0.99
Meniere’s disease	2.62 (0.84–8.15)	0.10
**Multiple sclerosis**	**4.60 (1.71–12.34)**	**<0.01**
**Oesophageal stricture**	**1.04 (1.46–74.46)**	**0.02**
Parkinson’s disease	1.12x10^−7^ (0-inf*)	1.00
Peripheral vascular disease	1.69 (0.54–5.30)	0.37
**Pernicious anaemia**	**6.93 (3.42–14.03)**	**<0.01**
Polycystic ovary syndrome	6.40 (0.89–46.21)	0.07
Prostate diseases	0.70 (0.37–1.31)	0.26
Psoriasis/eczema	0.74 (0.40–1.34)	0.323
Stomach ulcer	1.70 (0.80–3.60)	0.16
Thyroid diseases	0.77 (0.46–1.28)	0.31
Viral hepatitis	1.56 ×10^−7^ (0-inf*)	0.99
LTC count	1.05 (0.96–1.15)	0.12

Confidence interval (CI).

Statistically significant long-term conditions (LTCs) are shown in bold.

Models were adjusted for age, sex, socioeconomic status, BMI, level of physical activity, alcohol consumption and smoking status.

Statistical significance was indicated from a *p*-value of <0.05.

*Infinite coefficients.

**Table A3.** Multicollinearity assessment for the Survival Models.

Survival model with oesophageal cancer as outcome variable	
Predictor variable	VIF value
Age	1.09
Sex	1.78
Townsend score	1.11
Body Mass Index	1.21
Smoking status	1.42
Alcohol consumption amount	1.20
Smoking in pack years	1.43
Long-term condition count	2.63
Dementia	1.00
Psychoactive substance misuse	1.00
Alcohol problem	1.20
Parkinson’s disease	1.02
Chronic liver disease	1.03
COPD	1.11
Pernicious anaemia	1.01
Epilepsy	1.01
CHD	1.17
Diabetes	1.23
Bronchiectasis	1.02
Hypertension	1.46
Multiple sclerosis	1.00
Asthma	1.17
Psoriasis/eczema	1.05
Anxiety	1.01
Painful conditions	1.18
Diverticular disease	1.02
Migraine	1.02
Barrett’s oesophagitis	1.05
Stomach ulcer	1.02
H. pylori infection	1.00
Hiatus hernia	1.07
**Survival model with stomach cancer as outcome variable**	
Predictor variable	VIF value
Age	1.11
Sex	1.22
Townsend score	1.09
Body Mass Index	1.17
Smoking status	1.44
Alcohol consumption amount	1.18
Smoking in pack years	1.47
Long-term condition count	1.59
Anxiety	1.02
Eating disorders	1.03
Atrial fibrillation	1.03
Barrett’s oesophagitis	1.01
Bronchieactasis	1.00
Chronic fatigue syndrome	1.02
CHD	1.20
COPD	1.05
Duodenal ulcer	1.00
Gastritis	1.00
Glaucoma	1.02
Heart failure	1.00
Pyloric stenosis	1.00
Meniere’s disease	1.01
Multiple sclerosis	1.01
Oesophageal stricture	1.00
Parkinson	1.00
Peripheral vascular disease	1.02
Pernicious anaemia	1.04
Polycystic ovarian syndrome	1.02
Prostate disease	1.03
Psoriasis/eczema	1.02
Stomach ulcer	1.02
Thyroid disease	1.07
Viral hepatitis	1.00

VIF = variance inflation factor.
